# Conformational change-based electrochemical biosensor for the simple and highly selective detection of miRNA in whole serum

**DOI:** 10.1039/d5ra06247k

**Published:** 2025-10-23

**Authors:** Hedieh Haji-Hashemi, Kandeel Shafique, Beatriz Prieto-Simón

**Affiliations:** a Institute of Chemical Research of Catalonia, The Barcelona Institute of Science and Technology Av. Països Catalans, 16 43007 Tarragona Spain bprieto@iciq.es hhaji@iciq.es; b Universitat Rovira i Virgili 43007 Tarragona Spain; c ICREA Pg. Lluís Companys 23 08010 Barcelona Spain

## Abstract

MicroRNAs (miRNAs) have emerged as powerful biomarkers for early-stage disease detection and prognosis support. However, their reliable quantification in complex biological fluids remains a significant challenge. Here, we present a simple and robust electrochemical DNA (E-DNA) sensor for the direct detection of miRNA-29c in whole human serum. The sensing mechanism harnesses the conformational change that occurs upon miRNA hybridization to the immobilized DNA capture probe. By exploiting the intrinsic base-pairing specificity of nucleic acids, our system achieves high selectivity, while the conformational change-based sensing mechanism confers resistance to electrode fouling. This dual advantage enables sensitive miRNA detection in complex media and effective discrimination of closely related miRNA sequences. Under optimized conditions, the biosensor exhibited a sigmoidal response to miRNA-29c in whole serum across the concentration range of 0.1–100 nM and showed excellent agreement with the Langmuir–Hill model (*R*^2^ = 0.994). Notably, the sensor demonstrated outstanding recovery rates (±10%) when challenged with serum spiked with known miRNA-29c concentrations. Furthermore, it displayed high selectivity and specificity in serum, showing significantly lower responses to non-complementary and two-base-mismatched sequences. Overall, the platform enables reagentless, amplification-free, and direct detection of miRNA in complex matrices such as whole serum, while maintaining high accuracy, reproducibility, and resistance to fouling. These findings underscore the efficacy of the conformational change-based electrochemical sensing approach, offering a promising avenue for practical, point-of-care miRNA diagnostics in clinically relevant settings.

## Abstract 2

Direct and selective miRNA-29c detection in undiluted human serum enabled by combining nucleic acids base-pairing specificity and conformational change-based sensing.

## Graphical abstract

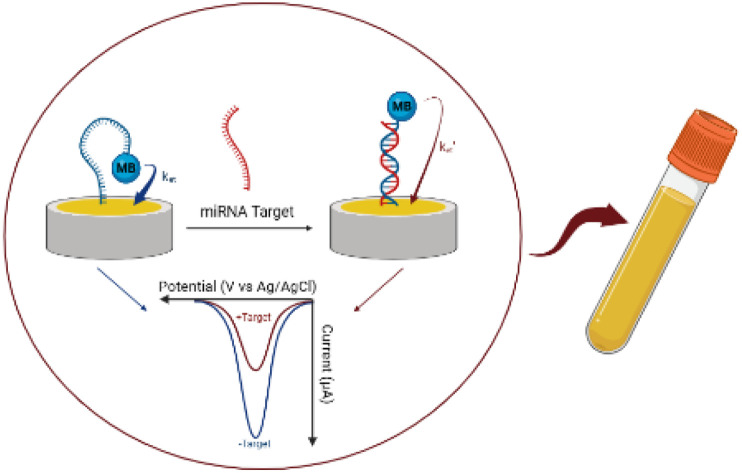

## Introduction

MicroRNAs (miRNAs) are small, non-coding, single-stranded RNA sequences of approximately 20–24 nucleotides found both intracellularly and extracellularly in plants, animals and humans.^1^ These molecules regulate critical cellular processes including division, differentiation, apoptosis, and other physiological or pathological functions.^2^ In addition to those functions, in recent years, it has been shown that dysregulation of circulating and exosomal miRNAs is correlated with several diseases, ranging from cancers to infectious diseases, making them promising diagnostic and prognostic biomarkers.^3^ Distinct miRNA expression signatures have demonstrated their potential not only for early-stage disease diagnosis but also for monitoring the progression of advanced-stage diseases, prognosis prediction, and medication resistance.^4^

Despite their attractiveness as biomarkers, the clinical implementation of miRNA detection faces several challenges. The primary challenge is the low and often unknown levels of miRNAs in biological fluids.^1^ Moreover, miRNAs are short sequences with high homology that must be detected within complex biological media.^5^ As a result, the ideal technique for miRNA detection must provide high sensitivity, specificity, and robustness to enable accurate quantification directly in biofluids tackling such challenging conditions. Current miRNA detection techniques, such as northern blotting,^6,7^ microarray analysis,^8,9^ quantitative polymerase chain reaction (qPCR),^10^ and next-generation sequencing^11^ are accurate but face significant limitations. They typically rely on miRNA extraction from biological fluids and/or amplification steps and, in practical scenarios, multiple costly and labor-intensive techniques are often combined to improve accuracy.^12^ These requirements complicate the workflow and limit direct testing in complex biofluids, reducing the feasibility of routine clinical use. To address these challenges, extensive efforts have been made over recent decades to develop new detection techniques that meet the needs for reliable, quantitative miRNA analysis.

In this regard, electrochemical biosensors have attracted significant interest for miRNA detection due to their affordability, ease of use, rapid response times, straightforward miniaturization, and compatibility with mass fabrication—attributes that make them particularly well-suited for point-of-care testing and the development of portable diagnostic devices.^13,14^ Typically, electrochemical miRNA biosensors rely on hybridization-based recognition. By harnessing the intrinsic base-pairing specificity of nucleic acids, these biosensors achieve accurate quantification of miRNA targets and effectively discriminate among closely related miRNA sequences.^15,16^ Notwithstanding the significant advantages and ongoing research progress, the practical application of this type of biosensor for miRNA detection in real biological fluids remains limited. Most studies have demonstrated miRNA detection in simplified matrices, such as buffer solutions,^17^ diluted biological fluids,^18,19^ or aqueous solutions of reconstituted RNA extracts,^20,21^ rather than directly in complex samples like undiluted serum, urine, blood, plasma, or saliva. This limitation arises primarily from the high susceptibility of the electrochemical signals to nonspecific adsorption and electrode fouling caused by the multitude of biomolecules present in complex matrices. Indeed, in most electrochemical biosensors fouling produces a signal that is often indistinguishable from that generated by target binding, substantially compromising performance by reducing both the signal gain magnitude and the biosensor's specificity.^22^ As an example, Ren *et al.* reported direct miRNA profiling in serum through an electrochemical miRNA assay with signal amplification triggered by nuclease cleavage of the hybridized probe, although results were consistently lower compared to those obtained when extracted RNA was analyzed, due to electrode fouling effects.^23^

A promising solution to the above challenge has been proposed by mimicking the monitoring mechanism found in nature, namely, binding-induced changes in the physical conformation of a receptor.^24^ Indeed, instead of relying on binding-associated changes in diffusion, charge, or other measurable properties, nature capitalizes on structural changes within the receptor itself to translate a binding event into a readily detectable signal. The application of binding-induced conformational changes in electrochemical biosensing is a relatively recent advancement.^25^ In this approach, the detection principle relies on a structural rearrangement that takes place upon target binding to a redox-tagged probe (mostly an oligonucleotide sequence specific to the analyte) that is site-specifically anchored to an interrogating electrode. Upon target binding, the resulting conformational change modulates the rate of electron transfer from the reporter tag.^26–28^ Because the signal change is structurally driven and requires the correct target to induce the conformational change, the biosensors based on this detection principle are largely insensitive to nonspecific adsorption, allowing for direct deployment in biological fluids both *in vitro* and *in vivo* for extended periods.^29^

This class of conformational change-based electrochemical biosensors can generally be divided into two main categories:^30^ electrochemical aptamer-based (E-AB) biosensors, where the probe is an artificially selected oligonucleotide sequence specific to a molecular target (*e.g.*, drugs, proteins, *etc.*),^31^ and electrochemical DNA (E-DNA) sensors, where the capture probe is a synthesized sequence complementary to a DNA or RNA target.^32^ Although E-AB biosensors have been extensively investigated and successfully applied to the direct detection of various targets, including drugs,^33,34^ antibiotics,^25,35,36^ and protein biomarkers,^37,38^ in complex matrices and even in the living body,^39^ the application of E-DNA sensors for DNA and RNA detection in undiluted real samples remains underexplored.

**Fig. 1:**
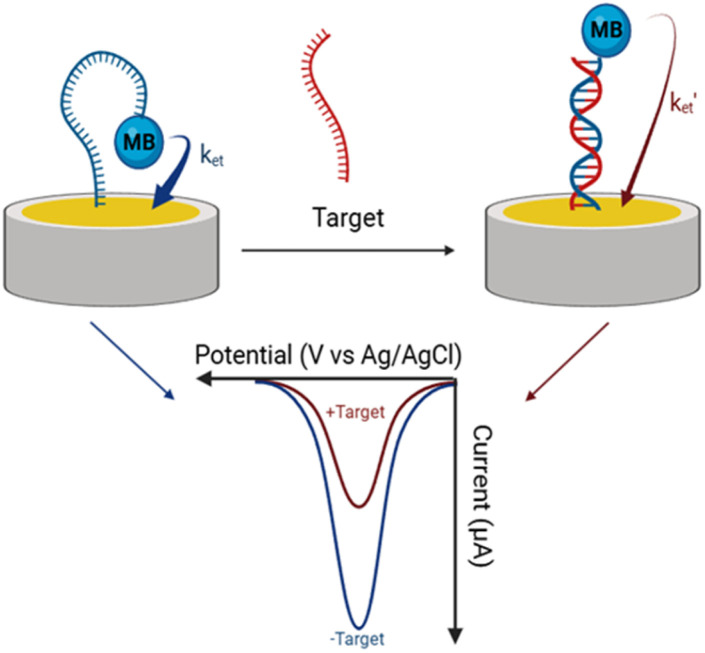
Schematic illustration of the E-DNA sensing approach.

Motivated by this gap and the growing need for reliable miRNA detection, the present work explores the feasibility of using E-DNA sensors for miRNA detection in complex biological matrices. As a proof of concept, we employed an E-DNA sensing platform to detect and quantify miRNA-29c directly in whole human serum. miRNA-29c is implicated in key cellular processes such as proliferation, differentiation, apoptosis, and DNA methylation.^40^ Moreover, elevated miRNA-29c levels in serum have been associated with early-stage pathological conditions such as colorectal cancer,^41^ non-small cell lung cancer,^42^ and atherosclerosis,^43^ underscoring its potential as a circulating biomarker for early disease diagnosis. Our sensing approach involves modifying gold electrodes with a thiolated, methylene blue (MB)-tagged oligonucleotide probe complementary to miRNA-29c. In the absence of the target, the structure of the probe positions the redox tag in close proximity to the electrode surface, generating a pronounced faradaic current measurable by square-wave voltammetry (SWV). Upon hybridization with miRNA-29c, the redox tag is displaced away from the electrode, significantly reducing the current and thus enabling its detection (Fig. 1). Our hypothesis is that, in addition to enabling sensitive miRNA-29c quantification in whole serum, this conformational change-based sensing mechanism allows the discrimination of closely related miRNA sequences with remarkable precision, thereby underscoring its potential for highly specific and accurate diagnosis.

## Experimental

### Reagents and materials

All chemicals were purchased from Sigma-Aldrich Co. and used as received, unless otherwise noted. All aqueous solutions were prepared using Milli-Q water (from a Milli-Q Direct purification system, resistivity = 18 MΩ cm). Phosphate buffered saline tablets were used to prepare a 10 mM phosphate buffer solution containing 137 mM sodium chloride and 2.7 mM potassium chloride (PBS, pH 7.4). Purified synthetic ssDNA and RNA sequences were purchased from Biosearch Technologies (UK). Upon receipt, all sequences were dissolved in nuclease-free water (obtained from Integrated DNA Technologies, Inc., Belgium) at a concentration of 100 μM, aliquoted, and stored at −20 °C. Table 1 shows the sequences of all synthetic oligonucleotides employed for E-DNA sensor fabrication and validation.

**Table 1:** Sequences of the synthesized oligonucleotides used in this work

Oligonucleotide	Sequence (from 5′ to 3′)
ssDNA capture probe	SH(CH_2_)_6_-TAACCGATTTCAAATGGTGCTA-MB
Target ssDNA (sequence equivalent to that of miRNA-29c)	TAGCACCATTTGAAATCGGTTA
Target RNA (miRNA-29c)	UAGCACCAUUUGAAAUCGGUUA
Non-complementary RNA 1 (NC1)	UGAGAACUGAAUUCCAUAGGCUGU
Non-complementary RNA 2 (NC2)	UGU CAG UUU GUC AAA UAC CCC
Non-complementary RNA 3 (NC3)	AGCUACAUUGUCUGCUGGGUUU
2-Base mismatch RNA (2bmm)^a^	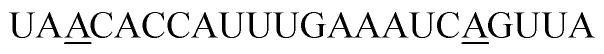

a Underlined bases denote the positions of the intentional mismatches.

It should be noted that in our preliminary experiments and throughout the optimization steps, we used a ssDNA target sequence corresponding to that of miRNA-29c instead of the RNA version, primarily due to DNA's lower cost, reduced susceptibility to degradation, and the elimination of the need for RNase-free conditions. Moreover, ssDNA serves as a model system that can readily be translated to RNA targets.^44,45^ Accordingly, the platform's protocols and working conditions were optimized using DNA. After finalizing these parameters, RNA targets were employed in the subsequent experiments.

### Electrochemical setup

All electrochemical measurements were performed at room temperature using an IVIUM CompactStat potentiostat (Ivium Technologies, Netherlands). The electrochemical cell setup included a three-electrode system containing a platinum counter electrode (TianjinAida Co., China), an Ag/AgCl reference electrode (TianjinAida Co., China), and a 2 mm diameter gold electrode (CH Instruments, Inc., Austin, TX, USA) as the working electrode.

### Biosensor fabrication

First, gold rod electrodes were sequentially polished on microcloth pads using 1 μm, 0.3 μm, and 0.05 μm alumina slurries (CH Instruments, Inc., Austin, TX, USA) for 5 minutes each. Next, the electrodes were sonicated for 5 minutes in 95% ethanol and dried under a nitrogen stream. They were then immersed in a piranha solution (a 1 : 3 mixture of 30% H_2_O_2_ and concentrated H_2_SO_4_) for 5 minutes, followed by thorough rinsing with Milli-Q water (caution: piranha solution is a powerful oxidizing agent and reacts vigorously with organic materials; it must always be handled under a fume hood with proper protective equipment). Next, the electrodes underwent electrochemical pretreatment by cycling the potential from −0.1 to 1.6 V *versus* an Ag/AgCl reference electrode at a scan rate of 0.1 V s^−1^ for 40 scans in 0.5 M H_2_SO_4_. The electrodes were then rinsed with Milli-Q water and dried under a stream of nitrogen. For the immobilization of the capture probe, an aliquot of the thiolated DNA probe sequence was thawed, and a 1000-fold molar excess of tris(2-carboxyethyl)phosphine (TCEP) was added, prior to incubating the solution for 1 hour at room temperature in the dark, to reduce any potentially formed disulfide bonds. The solution was then diluted to a final concentration of 500 nM using PBS. Cleaned gold electrodes were incubated in this solution for 2 hours, followed by a rinse with Milli-Q water. The electrodes were then incubated overnight at room temperature in 3 mM 6-mercaptohexanol (MCH) freshly prepared in PBS. After a final rinse with Milli-Q water, the biosensors were ready to use.

### Biosensor electrochemical response measurement

The electrochemical response of the biosensor was recorded using square wave voltammetry (SWV). SWV measurements were conducted within a potential window of −0.1 to −0.45 V, utilizing an amplitude of 0.025 V, a potential step of 0.003 V, and a frequency of 50 Hz. The signal gain for the E-DNA sensors was calculated using the following equation (eqn (1)):1
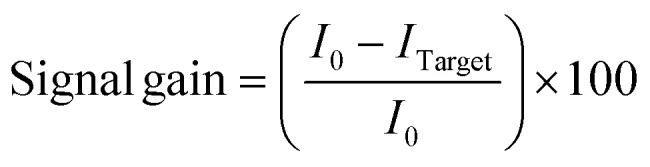
where *I*_0_ is the background current recorded in the absence of the target and *I*_Target_ represents the current measured in the presence of the target. For all measurements, freshly prepared biosensors were initially interrogated in PBS until a stable SWV baseline signal was established.

For the optimization study of the hybridization conditions, once the baseline signal stabilized, the biosensors were exposed to a PBS solution containing 0, 10 and 100 μM of the DNA target, sequentially. Incubation was conducted either by direct immersion in the solution (at ambient conditions) or by drop-casting 30 μL of the solution onto the electrode surface in a homemade humid chamber (maintained with boiling water to ensure high humidity). SWV measurements were recorded at 10-minute intervals in the corresponding incubation solution, until the signal gain stabilized for each concentration.

For subsequent measurements, once the baseline signal stabilized, the biosensors were first incubated with 30 μL of PBS in the humid chamber for 1 hour, and then with the target solutions under the same conditions. After each incubation step, SWV measurements were performed in the corresponding incubation solution. Fitting of titration curves was performed using KaleidaGraph software.

### Human serum samples

In serum and plasma, most circulating miRNAs are bound to various proteins, including nucleoplasmin, Argonaute2, and high-density lipoprotein (HDL). Consequently, a protein denaturation step is required to release miRNAs from these protein carriers and ensure accurate detection.^46^ Proteinase K treatment is widely employed in miRNA extraction protocols due to its efficiency in denaturing proteins and releasing bound miRNAs, thereby enhancing detection accuracy.^47–49^ In our experiments, we incorporated this step to prevent spiked miRNAs from binding to serum proteins. Specifically, human serum (male AB plasma, USA; sterile-filtered; Merck, H4522) was first centrifuged at 1500 rpm for 10 minutes, and the supernatant was collected, aliquoted, and immediately frozen. After thawing, the serum was mixed with 1% w/v SDS and proteinase K (Thermo Scientific, ST533) at a final concentration of 25 μg mL^−1^. Following a 30-minute incubation at room temperature, EDTA was added to a final concentration of 20 mM to inhibit proteinase K.^50^ Finally, the miRNA targets were spiked into this treated serum to achieve the desired concentrations.

### Statistical analysis

Statistical analysis was performed using GraphPad Prism. An unpaired *t*-test was used to compare the signal responses between different sample groups, with a confidence interval (CI) of 95% and statistical significance set at *p* < 0.05.

## Results and discussion

In this study, we aimed to develop a simple and robust electrochemical biosensor for detecting miRNA-29c in complex biological samples, specifically whole human serum. The sensor design relies on the conformational change of a DNA probe attached to a gold electrode. Upon target hybridization, the probe undergoes a structural rearrangement, altering the proximity of the redox tag (MB) to the electrode surface, which in turn generates a measurable change in the SWV signal. This sensing approach combines the base-pairing specificity of nucleic acids, which ensures high selectivity, with a conformational change-based transduction mechanism that resists electrode fouling. We hypothesized that this combination could effectively address two major challenges in miRNA detection: (i) direct quantification of miRNA in complex biological fluids, and (ii) high-specificity discrimination among closely related miRNA sequences. To explore this, we first established the fundamental electrochemical response of the sensor and optimized key experimental parameters, such as capture probe concentration, hybridization time, and incubation methodology, in simplified buffer conditions using a ssDNA sequence as the target complementary to the immobilized capture probe. We then demonstrated the sensor's performance in whole human serum, including its resistance to nonspecific interference and its ability to detect miRNA-29c spiked in human serum, underscoring its potential for clinical applications.

**Fig. 2:**
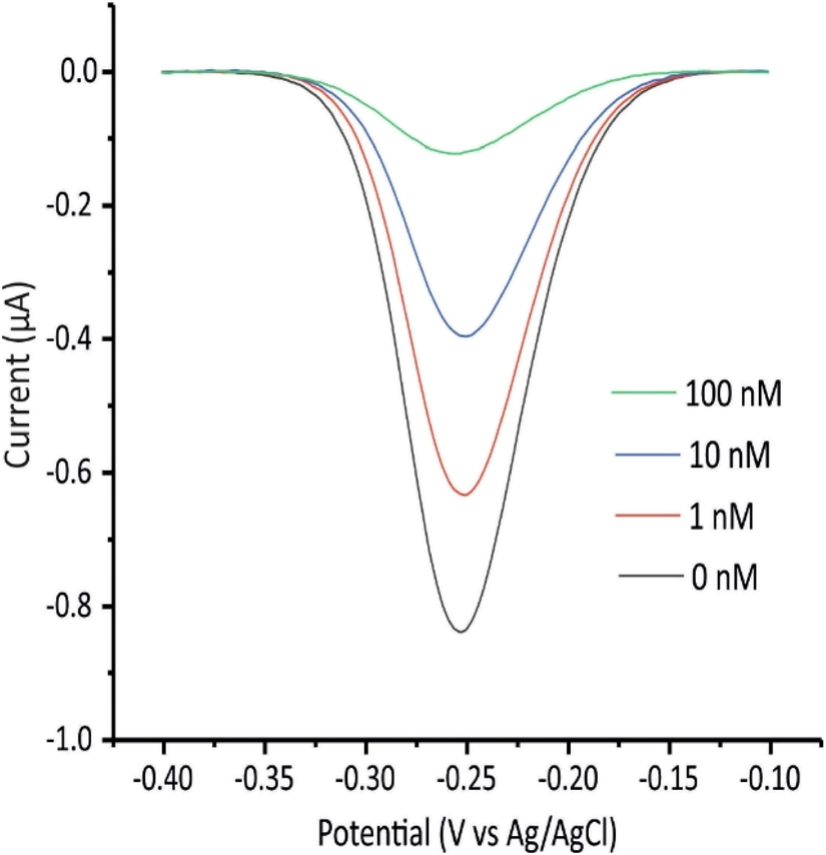
Square-wave voltammograms obtained using the E-DNA sensor upon incubation in PBS containing no target, 1, 10, and 100 nM of target DNA. Each measurement was recorded following 1 hour incubation in the humid chamber.

As an initial step, the fabricated biosensors were tested with increasing concentrations of the target DNA. Fig. 2 displays the square wave voltammograms obtained after incubating the biosensor in PBS without any target and in the presence of 1, 10, and 100 nM of target DNA. As expected, upon target hybridization, a monotonic decrease in current was observed, confirming the sensing ability of the platform. To confirm that the observed decrease in current originated from target hybridization rather than electrode fouling or probe degradation, regeneration of the biosensor was evaluated. A brief rinse in deionized water followed by incubation in PBS for a few minutes^51^ enabled nearly complete recovery of the baseline signal, demonstrating that the current change was due to hybridization (Fig. S1A). Importantly, the biosensor was reused seven consecutive hybridization cycles showing less than ∼5% signal loss, highlighting its stability and reusability (Fig. S1B).

**Fig. 3:**
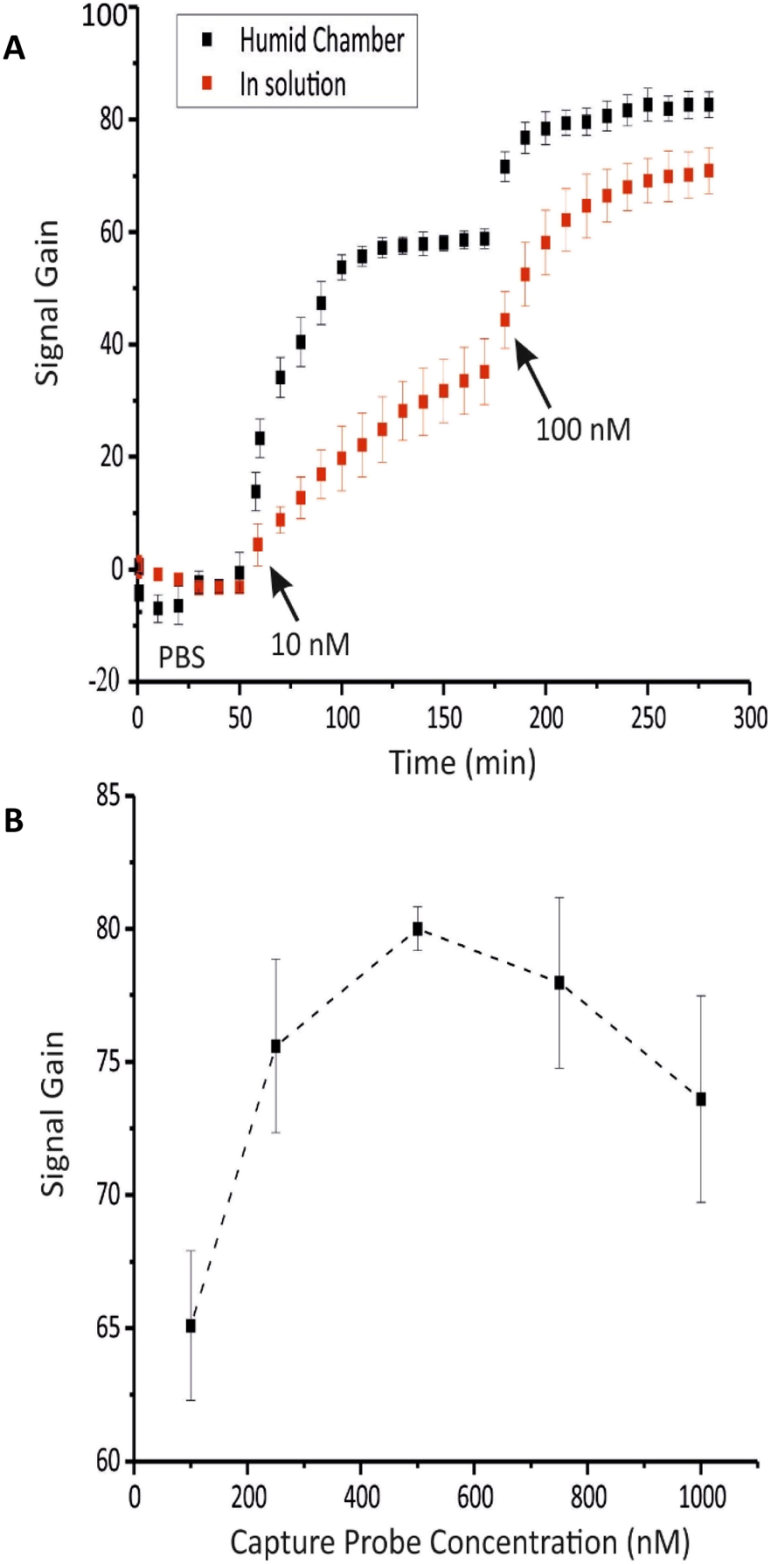
(A) Hybridization conditions optimization. Signal gain was recorded every 10 minutes after exposing the biosensors to PBS with no target and to 10 and 100 nM solutions of the target DNA prepared in PBS. Incubation in each solution was performed either at ambient conditions or in the humid chamber. (B) Capture probe concentration optimization. Biosensors were fabricated with capture probe concentrations of 100, 250, 500, 750, and 1000 nM, and the signal gain was measured in response to a 50 nM target DNA solution prepared in PBS. The illustrated values are the average signal gain values achieved from 3 individual biosensors, and error bars represent the standard deviation of these measurements.

Next, we investigated the influence of the hybridization conditions on the biosensor's performance, given their substantial impact on sensitivity. To this end, we compared two different approaches: hybridization at ambient conditions *versus* hybridization in a homemade humid chamber. Two sets of biosensors were incubated with 0, 10, or 100 nM target DNA solutions, prepared in PBS, using each method, and the biosensor response was measured every 10 minutes until the signal stabilized. As shown in Fig. 3A, when using the solution-based approach, the maximum signal gains for 10 nM and 100 nM targets were approximately 35% and 70%, respectively. In contrast, these values increased to 57% and 82% with the humid chamber method, which also led to faster stabilization of the signal (*e.g.*, at 10 nM target concentration, the signal plateaued within one-hour incubation in the humid chamber *versus* approximately two-hour incubation at ambient conditions). It is worth mentioning that the response time observed in this work is consistent with values reported for comparable solid-phase, hybridization-based DNA biosensors.^52–54^ The enhanced and fast response of the humid chamber method is likely due to the slightly elevated temperature applied, which promotes DNA mobility and accelerates hybridization kinetics. Indeed, the recorded temperature of the humid chamber over the one-hour incubation period (∼35 °C) was within the optimal range (below 40 °C) established in literature for nucleic acid hybridization in electrochemical sensing.^55^ Consequently, the humid chamber method with one-hour incubation was adopted for subsequent experiments to ensure maximum hybridization efficiency.

Additionally, the concentration of the capture probe was optimized by preparing biosensors with varying probe concentrations (100, 250, 500, 750, and 1000 nM) and then recording their signal gain in response to a 50 nM DNA solution prepared in PBS. As shown in Fig. 3B, the signal gain increased with rising capture probe concentration, peaking at 500 nM before gradually declining at higher concentrations. This trend can be attributed to insufficient surface coverage at low probe concentrations, which limits hybridization efficiency, and to excessive electrostatic repulsion coupled with steric hindrance at high probe densities, both of which impede effective hybridization.^56^ Consequently, 500 nM was used as optimum capture probe concentration for subsequent experiments.

**Fig. 4:**
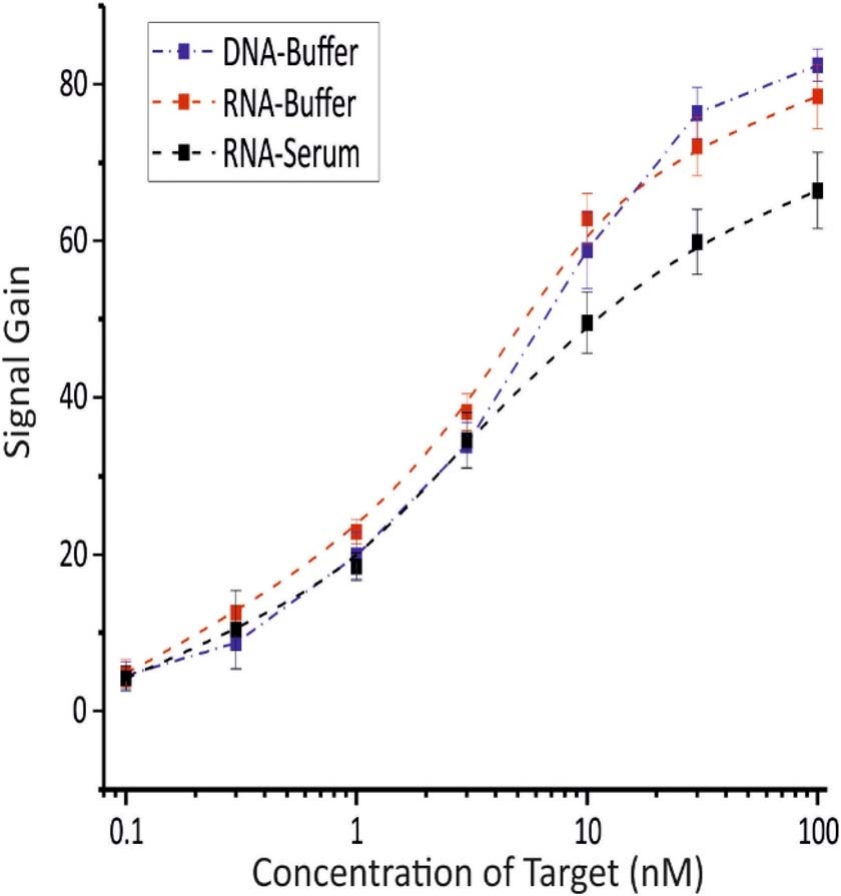
Titration curves obtained from the biosensor in response to target DNA in buffer (blue), miRNA in buffer (red), and miRNA in serum (black). Signal gain was measured after 1 hour incubation in a humid chamber, with different target concentrations ranging from 0.1 to 100 nM. The illustrated values are the average signal gain achieved from 3 individual biosensors, and error bars represent the standard deviation of these measurements.

Under the optimal conditions, the analytical performance of the biosensor was initially evaluated by detecting various concentrations of both DNA and miRNA targets in PBS, using two distinct sets of biosensors. As presented in Fig. 4, the titration curves for both DNA and miRNA targets exhibited the expected sigmoidal trend characteristic of such binding events within the range of 0.1–100 nM, ultimately reaching a similar maximum signal gain of approximately 80% for each target (85.5 ± 2.3 for DNA and 79.6 ± 1.3 for miRNA). The titration curves were well-fitted to the Langmuir–Hill equation (Fig. S2 and S3) with *R*^2^ values of 0.994 and 0.998 for DNA and miRNA, respectively. The apparent dissociation constants (*K*_D_) of 4.12 ± 0.46 nM for DNA and 2.71 ± 0.19 nM for miRNA were derived from this fitting, indicating that the labeled DNA capture probe exhibits slightly stronger binding affinity for RNA than for DNA. This observation aligns with existing literature, where DNA/RNA heteroduplexes are reported to be thermodynamically more stable than DNA/DNA duplexes.^57^

In the next step, the biosensor's applicability for detecting miRNA-29c in complex biological matrices was evaluated by detecting various concentrations of miRNA-29c into whole serum. As shown in Fig. 4, the resulting titration curve exhibited a similar sigmoidal trend to that observed in PBS over the 0.1–100 nM range, reaching a maximum signal gain of 66.3 ± 1.7%, 10% lower than the value recorded in PBS. Moreover, the Langmuir–Hill equation provided an excellent fit (*R*^2^ = 0.994) and yielded a *K*_D_ of 2.62 ± 0.29 nM, closely matching the value obtained in PBS (Fig. S4).

A practical quantitation limit (PQL) of 0.1 nM miRNA-29c, both in PBS and whole serum, was experimentally determined as the lowest analyte concentration that provided acceptable precision in the calculated signal gain (RSD < 10%) based on replicate measurements.

In addition, it is worth noting that the baseline current of the biosensor recorded in treated serum was essentially equal to that measured in PBS (Fig. S5). The negligible effect of serum on the amplitude of the measured MB signal, combined with the comparable signal gain and PQL values obtained in both media (Fig. S6), further support our hypothesis that reliable and direct detection of miRNAs in complex biological fluids is possible by harnessing sensing mechanisms based on probe conformational changes.

To further evaluate our initial hypothesis of enabling direct miRNA quantification in complex biological fluids, we assessed the biosensor's ability to measure unknown miRNA-29c levels in serum. In this experiment, the titration fit obtained for miRNA-29c in serum was used to estimate the recovered concentrations measured by an additional set of biosensors that were not part of the initial titration. As shown in Table 2, the biosensor achieved excellent analytical accuracy in measuring unknown miRNA-29c levels in serum, with recovery rates consistently within ±10% of the spiked miRNA concentrations across the 0.1–100 nM range. These results underscore the excellent performance of this simple conformational change-based biosensor for miRNA detection and quantification in complex media (*e.g.*, serum), effectively overcoming challenges such as electrode fouling and nonspecific binding.

**Table 2:** Recovery rates of the developed E-DNA sensor detecting different miRNA-29c concentrations spiked in treated serum

miRNA-29c concentration (nM)	Recovery%	SD
0.1	104.4	3.8
1	98.9	1.7
10	102.2	2.3
100	94.8	1.3

**Fig. 5:**
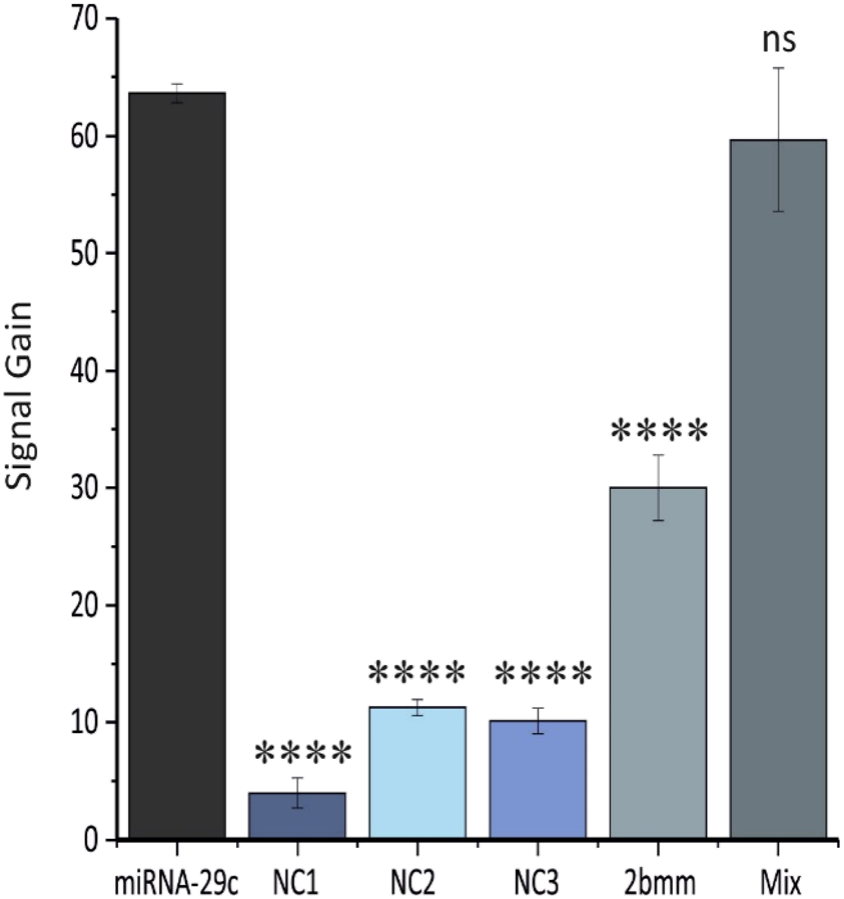
Selectivity results of the biosensor. Signal gain was recorded in treated serum samples containing three non-complementary (NC) miRNAs, or a two-base mismatched RNA sequence, all at 100 nM, and in a treated serum solution containing miRNA-29c together with all three NC sequences (100 nM each). The obtained results were compared to the signal gain recorded for miRNA-29c at 100 nM in serum. The illustrated values are the average signal gain values achieved from 3 individual biosensors, and error bars represent the standard deviation of these measurements. Statistical significance was determined by unpaired *t* test (significance code: ****p ≤ 0.0001, ns = not significant).

To evaluate our second hypothesis of achieving high selectivity and specificity in distinguishing closely related miRNA sequences in complex matrices, we recorded the biosensor's response in serum to three non-complementary (NC) miRNAs and a two-base mismatched (2bmm) RNA sequence, each at 100 nM, and compared these responses to that of the target miRNA-29c at 100 nM. As shown in Fig. 5, the responses to the three NC RNAs and the 2bmm RNA sequence were significantly lower than that to the target miRNA-29c, demonstrating excellent selectivity and specificity. Furthermore, we assessed the biosensor's performance in the presence of potential interferences by exposing it to a mixture containing miRNA-29c (100 nM) together with all three NC sequences (100 nM each) in serum. Notably, the signal obtained from this mixture was not significantly different from that recorded for the target miRNA-29c alone, confirming robust detection capability even in the presence of interferences. These results underpin the ability of the developed E-DNA sensor to precisely differentiate closely related miRNA sequences in complex matrices, a critical requirement for reliable clinical diagnostics.

## Conclusions

In this study, we developed and demonstrated a simple yet robust E-DNA sensor leveraging a conformational change-based sensing mechanism for the direct detection and quantification of miRNA-29c in whole human serum. This conformational change-based approach provides significant advantages, effectively addressing critical challenges associated with existing miRNA detection methods, such as the requirement for extraction and amplification steps, and susceptibility to electrode fouling and nonspecific adsorption caused by the multitude of biomolecules present in complex biological fluids. Under optimized experimental conditions, the developed E-DNA sensor showed a well-defined sigmoidal response to miRNA-29c concentrations ranging from 0.1 to 100 nM in treated serum, with excellent fitting to the Langmuir–Hill model and recovery rates consistently within ±10% of the spiked values. Notably, the platform exhibited remarkable specificity, clearly distinguishing closely related miRNA sequences and maintaining high accuracy even in the presence of interfering substances.

These results confirm our initial hypothesis, demonstrating that the conformational change-based E-DNA sensing mechanism uniquely combines accuracy with specificity, allowing reliable direct detection of miRNA-29c in undiluted serum samples. The obtained data further highlight the platform's potential for addressing existing knowledge gaps related to the absolute quantification of miRNAs in clinical samples, an essential step towards understanding their biological relevance and diagnostic utility. Given its inherent simplicity and excellent analytical performance, this biosensor platform holds great promise for integration into portable, point-of-care diagnostic devices. Future developments, including miniaturization, multiplexing capabilities, and clinical validation, could significantly enhance its applicability for early disease diagnosis and personalized therapeutic monitoring.

## Author contributions

H. H. H. and K. S. contributed equally to this work. H. H. H. and K. S. performed all sensor measurements, contributed to experimental design, and carried out data analysis. H. H. H. and B. P. S. collaboratively conceived and supervised the research. All authors participated in drafting the manuscript and revising it critically for important intellectual content, and they approved the final version for publication. B. P. S. was responsible for funding acquisition and project administration.

## Conflicts of interest

There are no conflicts to declare.

## Supplementary Material

RA-015-D5RA06247K-s001

## Data Availability

The raw data presented at this work (Excel File) and the fitting results are available at OSF *via* our view-only project link: https://osf.io/e7csu/?view_only=fd22f9f3778c446387d0354ddd839fb3. Supplementary information: additional experimental details and results, including: protocols for biosensor regeneration and reuse, and electrochemical data associated, non-linear regression analyses of titration curves using Langmuir-Hill isotherm, and electrochemical baseline measurements and biosensor response in buffer and serum. See DOI: https://doi.org/10.1039/d5ra06247k.
