# Supplementary material for: Conformational change-based electrochemical biosensor for the simple and highly selective detection of miRNA in whole serum
Source: RSC Adv. 2025 Oct 23;15(48):40469–77. doi: 10.1039/d5ra06247k (PMC12548363; doi:10.1039/d5ra06247k)
Supplement: RA-015-D5RA06247K-s001 [file RA-015-D5RA06247K-s001.pdf]

Supplementary Information (SI) for RSC Advances.
This journal is © The Royal Society of Chemistry 2025

Supporting Information

Conformational Change-based Electrochemical Biosensor for the Simple and Highly Selective Detection of miRNA in Whole Serum

Hedieh Haji-Hashemi†[1*], Kandeel Shafique†[1,2], Beatriz Prieto-Simón[1,3*]

[1] Institute of Chemical Research of Catalonia, The Barcelona Institute of Science and Technology, Av. Països Catalans, 16, 43007, Tarragona, Spain

[2] Universitat Rovira i Virgili, 43007, Tarragona, Spain

[3] ICREA, Pg. Lluís Companys 23, 08010, Barcelona, Spain

Table of Contents

Biosensor regeneration and reuse……………………………………………………...2

Titration curves fitting…………………………………………………………………...3

Baseline measurements and biosensor response in PBS and serum………………………5

†These authors contributed equally to this work.

Biosensor regeneration and reuse

To assess the efficiency in the regeneration of the biosensor, electrodes that had undergone hybridization with the target were rinsed with deionized water for a few seconds, followed by incubation in PBS for 2–3 minutes. To evaluate reusability, the regenerated biosensor was re-incubated in the humid chamber with 100 nM target DNA prepared in PBS for 1 hour. This hybridization/regeneration cycle was repeated in sequence along 7 cycles, showing less than ~5% signal loss.

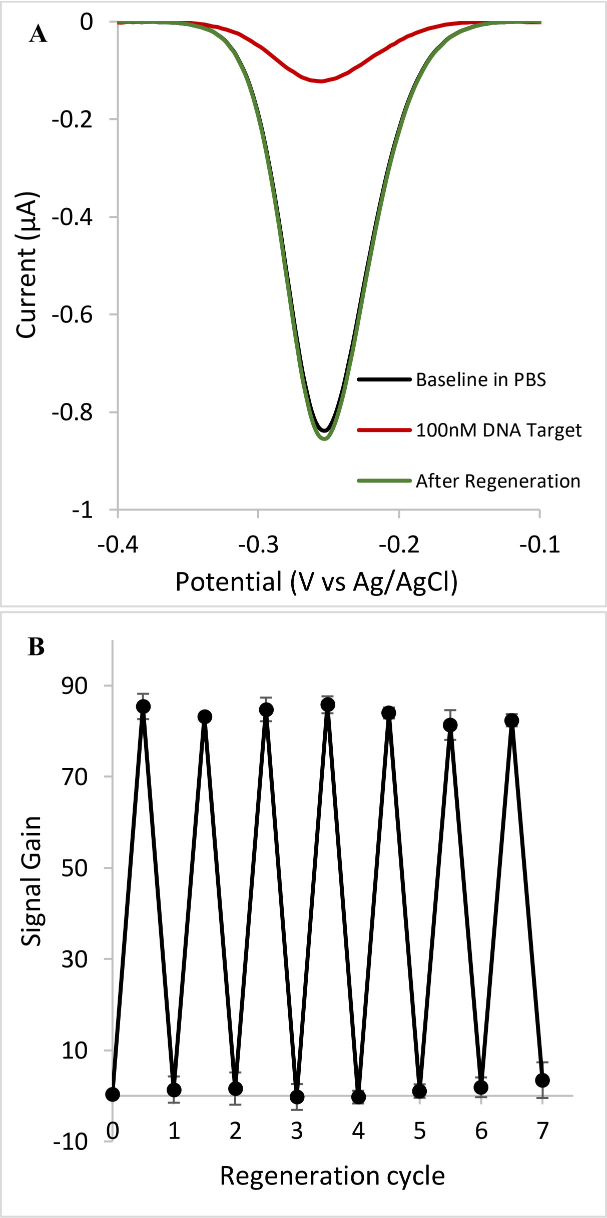

Figure S1. *(A) Square-wave voltammograms recorded upon incubation of the biosensor in PBS containing no target(black), 100 nM of target DNA (red), and after regeneration (grey). (B) Signal gain recorded over multiple consecutive hybridization/regeneration cycles, hybridization based on 1-hour incubation of biosensor in 100 nM DNA target solution prepared in PBS, and regeneration performed by 2–3 minutes biosensor incubation in PBS.*

Titration curve fitting

To determine the apparent sensor dissociation constants (K_D) and calculate the sensor recovery rates in response to miRNA-29c spiked in serum, we performed non-linear regression analyses using the Langmuir-Hill isotherm:

$$Signal\ Gain = \gamma \frac{[T]^n}{K_D + [T]^n}$$

where n is the number of aptamer binding sites (1 for all cases considered here), γ is the maximum signal output, and K_D is the apparent dissociation constant.[1] KaleidaGraph software was used for curve fitting, and Figures S2–S4 display the resulting fits.

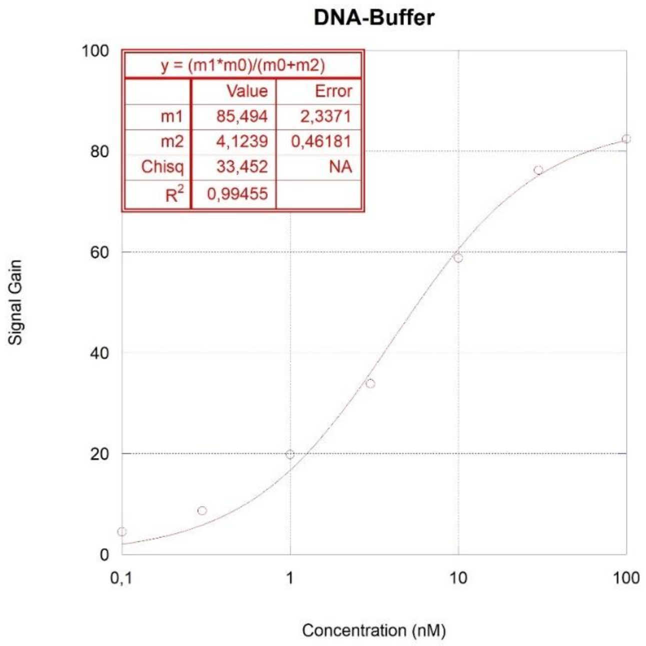

Figure S2. *Langmuir-Hill isotherm fit of sensor response data for target DNA detection in buffer.*

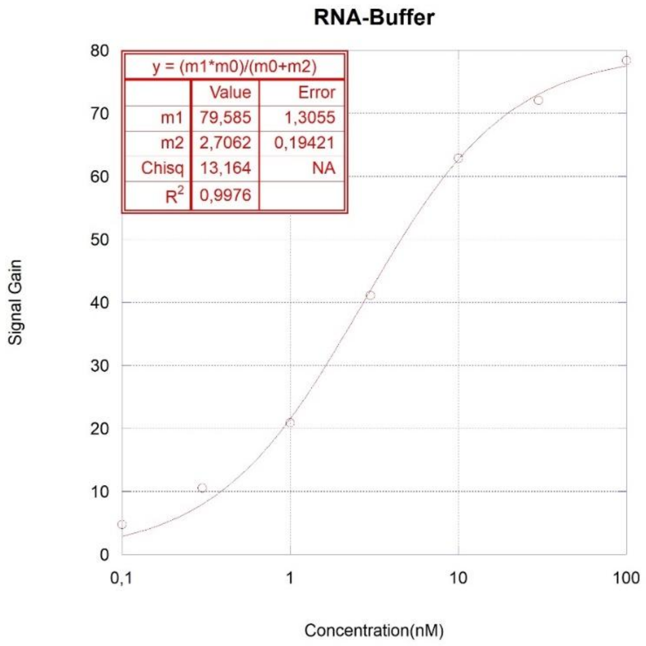

Figure S3. *Langmuir-Hill isotherm fit of sensor response data for miRNA-29c detection in buffer.*

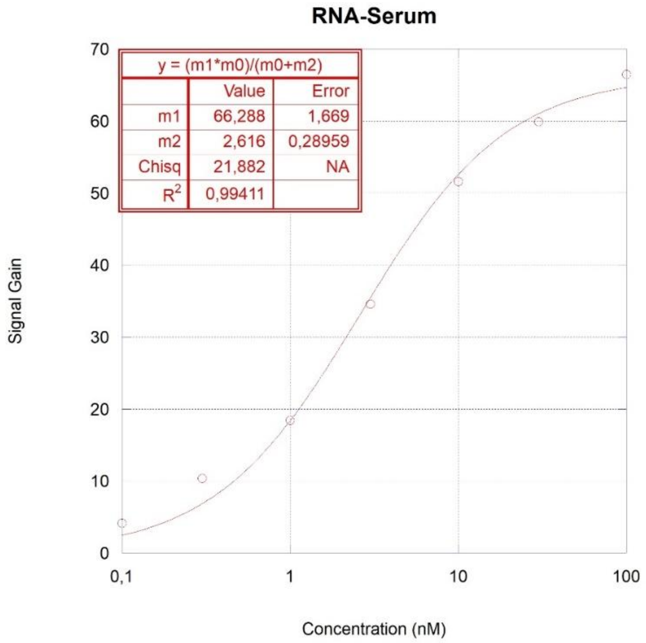

Figure S4. *Langmuir-Hill isotherm fit of sensor response data for miRNA-29c detection in human serum.*

Baseline measurements and biosensor response in PBS and serum

To evaluate the effect of the measurement medium on the electrochemical behavior of the biosensor, SWV measurements were performed using the same biosensor in three different conditions: (i) PBS, (ii) treated serum, and (iii) after 1 h incubation in treated serum in the humid chamber, all in the absence of target. As shown in Figure S5, the reduction currents recorded in PBS and serum were very similar, with only a negligible decrease observed after 1 hour incubation in serum. This result indicates that neither performing measurements in treated serum nor incubating the biosensor in this medium significantly affected the amplitude of the methylene blue (MB) reduction signal. The main difference observed between PBS and serum was a small positive shift in the MB reduction peak potential (from approximately -0.26 V in PBS to -0.22 V in serum). This shift can be attributed to changes in the interfacial environment of the tethered redox reporter when moving from a simple electrolyte to a complex biological matrix. In particular, treated serum contains anionic species (e.g., SDS, EDTA) and residual biomolecules that alter the composition of the electrical double layer and modify the local electrostatic environment of the tethered MB reporter. Such effects can stabilize MB and lead to a small positive shift in the apparent redox potential, while leaving the peak current unchanged. Similar redox potential shifts of surface-confined MB in different electrolytes and biological media have been reported and attributed to double-layer composition and specific interactions.[2] Importantly, this effect does not compromise the biosensor's quantitative performance. As shown in Figure S6, only a slight decrease in response was observed in serum compared to PBS, which can be attributed to the higher viscosity of serum, reducing the diffusion of the target toward the capture probe at the electrode surface.

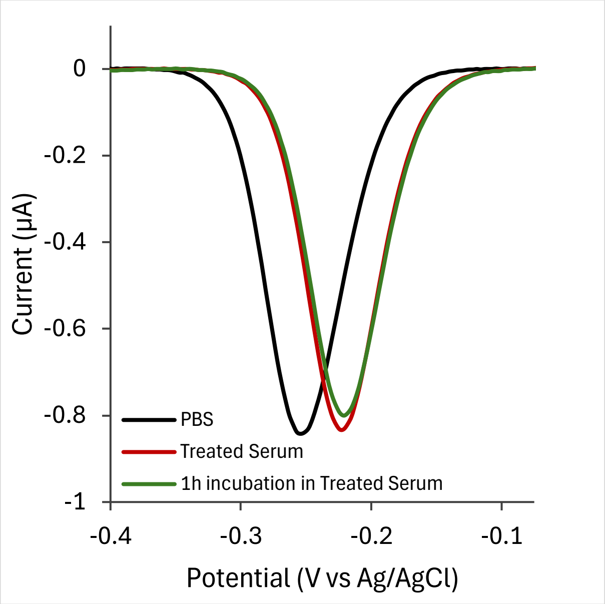

Figure S5. *Square-wave voltammograms recorded for a biosensor in PBS (black), treated serum (red), and after 1-hour incubation in treated serum inside the humid chamber (green).*

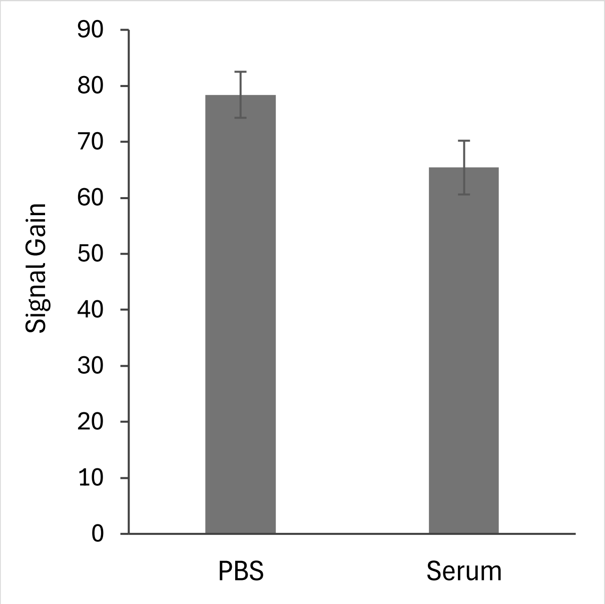

Figure S6. *Biosensor response to a 100 nM solution of miRNA-29c prepared in PBS and treated serum.*

References:

1. Wu Y, Tehrani F, Teymourian H, Mack J, Shaver A, Reynoso M, et al. Microneedle Aptamer-Based Sensors for Continuous, Real-Time Therapeutic Drug Monitoring. Anal. Chem. 2022;94:8335–45

2. Boon EM, Ceres DM, Drummond TG, Hill MG, Barton JK. Mutation detection by electrocatalysis at DNA-modified electrodes. Nat. Biotechnol. 2000;18:1096–100. https://doi.org/10.1038/80301
